# Real Estate Tax Base Assessment by Deep Learning Neural Network in the Context of the Digital Economy

**DOI:** 10.1155/2022/5904707

**Published:** 2022-08-09

**Authors:** Qiao Fu

**Affiliations:** Accounting School of Chongqing University of Technology, Chongqing University of Technology, ChongQing 400054, China

## Abstract

With the continuous development of China's digital economy and the continuous heating of the real estate market, real estate tax base assessment occupies an important position in the real estate market. The purpose is to improve the work efficiency of relevant personnel of real estate tax base assessment, reduce workload pressure, and improve the evaluation level. Real estate tax base assessment and real estate appraisal are studied in detail, and the factors of the real estate tax base assessment index are analyzed. Different real estate tax base assessment methods are compared, and the difference and connection between different methods are explored. The theory of batch assessment of real estate tax base is analyzed in depth, and the procedures for batch assessment implementation are summarized. On this basis, a deep learning neural network (DLNN) theory is proposed, and a real estate tax base assessment model based on DLNN is constructed. The reliability, accuracy, and relative superiority of the model are analyzed in detail, and the model is used to test the sample data and analyze the error. The results reveal that the DLNN model has better data fit and good reliability. Compared with other algorithms, it has certain advantages and smaller error values. In the sample test, the test value is closer to the actual value, the error is controllable, and it has high accuracy. Through training, it shows that the DL model has an excellent performance in tax base assessment, can meet the requirements of efficient batch assessment, and is expected to achieve the goal of completing a huge workload in a limited time and improve work efficiency. The real estate tax base assessment model by DLNN can bring some help to the real estate finance and taxation work and provide a reference for the batch assessment of tax base in the real estate industry.

## 1. Introduction

With the constant development of China's real estate market and the continuous progress of fiscal and taxation reforms, tax base assessment is a very important supporting measure for real estate tax management reform. The implementation of tax base assessment is conducive to the continuous regulation of real estate developers' tax behavior, which can ensure that real estate tax abides by certain standards and is beneficial to improving tax credibility [1]. As the stabilizer of China's economy, the development of the real estate market and the progress of China's economy are closely linked to each other and belong to the same strain [2]. With the rapid expansion of the digital economy, the real estate market will continue to heat up, and research on the real estate market is also a hot topic. The healthy development of real estate and the fairness of market transactions are inseparable from the assessment of the real estate tax base [3]. The study of real estate tax base assessment is of great significance to the real estate industry.

China's real estate tax base assessment is mainly based on a single assessment by the market method, cost method, and income method. However, there are many problems with this method. There are many commercial real estate transactions. It takes a lot of time to conduct a series of complex procedures such as field surveys, market research, and data collection during the evaluation [4]. The workload of the appraiser becomes very large, the error is large, and the evaluation efficiency is very low, which is not suitable for the trend of the development of the digital economy. To solve this problem, relatively speaking, the error results between the batch assessment and the actual transaction price are relatively satisfactory, so batch assessment has been widely used in China's real estate tax base assessment [5]. At present, there is a relatively mature real estate tax base assessment system with batch assessment as the core and tax assessment as the main purpose in the world [6]. However, the domestic evaluation system has not been perfected and needs further research, which has certain development potential. China's real estate value evaluation theory and practice research began with the reform and opening up. With the passage of time, China's cities have changed rapidly, and the factors affecting real estate value have become more diverse, and they affect each other, showing a complex nonlinear relationship [7]. Traditional evaluation methods have been unable to keep up with the changes in real estate development, and Internet technology has been gradually introduced into real estate value evaluation [8]. The deep learning (DL) model has high accuracy in the batch assessment of big data, and the technology is relatively mature and widely used. Chen et al. used the Deep Neural Networks (DNN) model to design an evacuation plan for subway station buildings and conducted simulation experiments. The accuracy and training speed of the model algorithm are verified by comparing with the convolutional neural network (CNN) model and the pre-training model of the classification dataset [9]. Li et al. proposed a multimodal medical image fusion method with DL according to the actual needs of medical diagnosis, the characteristics of multimodal medical images, medical diagnosis technology, and actual implementation to solve different types of multimodal medical image fusion problems [10]. Chen et al. used the DL model to design a network security system for the smart city and used this method to reduce network security risks [11]. Lv and Qiao combined cognitive computing and the Deep Belief Network (DBN) algorithm with collaborative robots to build a DBN-based cognitive computing system model and applied it to the control system of collaborative robots [12]. Zhang et al. put forward network intrusion detection (NIDS) method combining a traffic calculation model and a DL model for the real-time detection of high-speed network intrusions [13]. Although there are many studies, they mainly focus on the fields of computer algorithms, medicine, image recognition, and artificial intelligence (AI) integration technology, with less research on real estate appraisal.

To sum up, on account of the relevant literature, the method of combining theoretical research and experimental demonstration is adopted. The process and model of tax base assessment in real estate prices are designed by using the deep learning neural network (DLNN) model, and the feasibility and accuracy of the model are studied and analyzed. It is expected to provide a reference for the research on batch assessment of real estate tax bases in the context of China's new era of the digital economy.

## 2. Research Theory and Scheme Design

### 2.1. The Concept of Real Estate Tax Base Assessment

A real estate appraisal is the behavior of appraisal professionals to evaluate the value of real estate at a certain point in time and in a certain state of power. The real estate tax base refers to a certain degree of taxation breadth involving the number of taxation objects. It is the economic basis and objective basis for taxation. The real estate tax base has dual connotations of quality and quantity. Its quality mainly defines the object of taxation, and its quantity stands for the calculation base of the tax payable [14]. Tax base assessment is required to be scientific, rigorous, fair, and impartial, and it should be assessed by an authoritative department established by the government to ensure the rationality of the results and the conformity of market prices [15]. Real estate tax base assessment is for the purpose of tax collection, with assessment as the premise, and within the scope permitted by law, and follows certain legal procedures to appraise the real estate whose specific value cannot be clarified [16].

**Figure 1:**
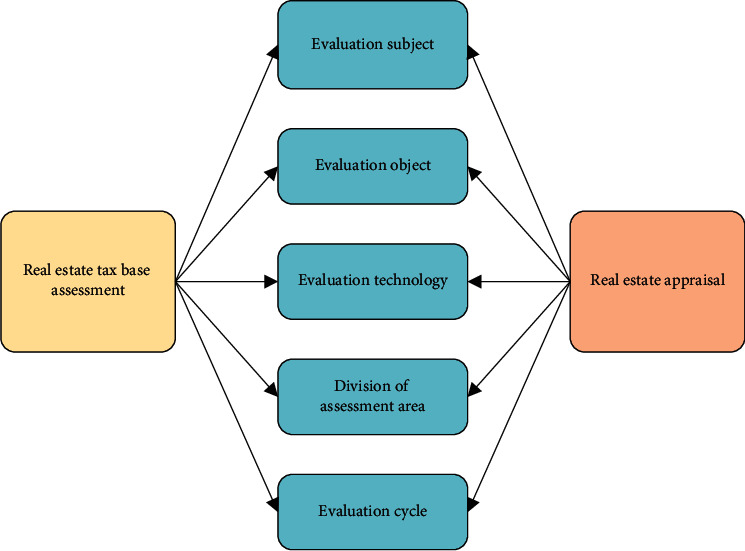
Similarities between tax base assessment of real estate and real estate appraisal.

There are many similarities between a real estate tax base assessment and a real estate appraisal. The specific overview is shown in Figure 1.

In Figure 1, the subjects of real estate tax base assessment and real estate appraisal are the undertakers of the assessment business, and real estate tax base assessment should highlight the functions and powers of the national government. In terms of assessment objects, real estate is used as the assessment object, but the real estate tax base assessment is for all real estate in the tax jurisdiction. A real estate appraisal is an activity on the strength of real estate prices at a certain point in time. In the aspect of evaluation technology, the support of relevant evaluation methods is required. In the division of assessment areas, the object of the real estate tax base is all taxable real estate within a region, which has a longer assessment period. Real estate tax base assessment must follow certain basic principles, including independence, objectivity, legality, highest and best use, substitution, assessment timing, contribution and expectation, and impartiality.

### 2.2. Factors of Real Estate Tax Base Assessment Index

**Figure 2:**
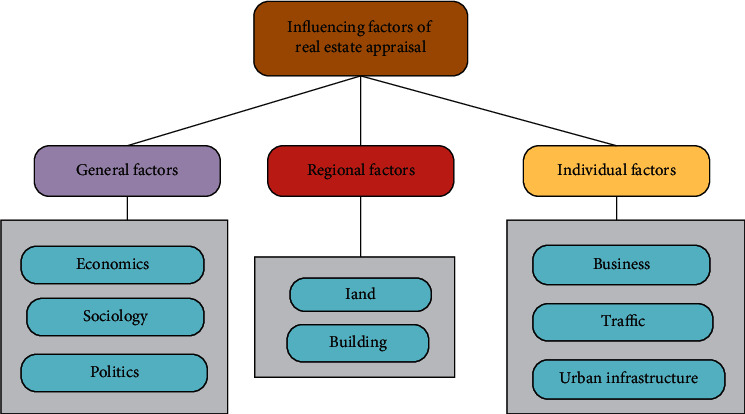
Influencing factors of real estate appraisal.

The real estate tax base assessment is based on the assessed value of the real estate market. Therefore, it is necessary to first understand the factors that affect the real estate value to select and determine the real estate tax base assessment index [17]. According to the classification of factors affecting real estate prices, it is mainly divided into general factors, regional factors, and individual factors [18]. Each factor is described in detail, as indicated in Figure 2.

In Figure 2, the general factors that affect the price of real estate refer to the general social and economic aspects that have a greater impact on the state and price level of real estate, comprising social factors, economic factors, and political factors. General factors determine the basis of real estate prices. Regional factors involve the geographical characteristics of the environment where real estate is located, including not only natural factors but also social factors. General factors involve a larger scope of influence, while regional factors have a smaller scope of influence than general factors, but they directly affect the real estate market. The individual factors that affect real estate prices refer to the influence on real estate prices due to the microscopic differences between real estate individuals. This is mainly determined by the uniqueness of real estate.

### 2.3. The General Method of Real Estate Tax Base Assessment

**Figure 3:**
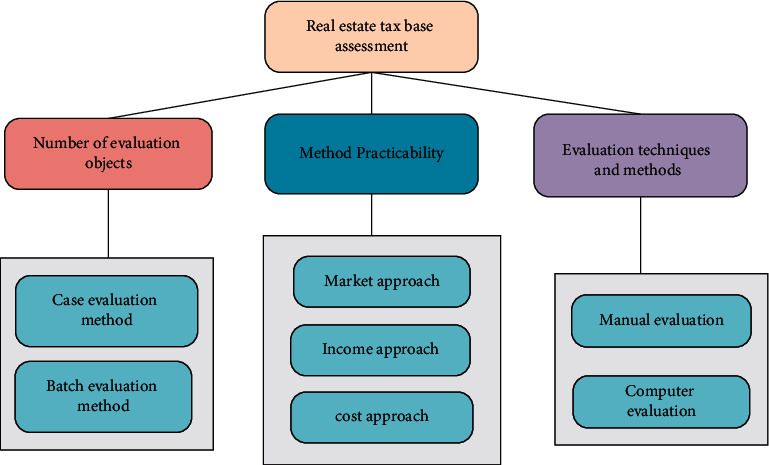
The general method of real estate tax base assessment.

There are many methods of real estate tax base assessment, and the assessment object, assessment technology, and assessment method are used as the basis for classification. Due to different classifications, the specific methods are also different [19]. The specific content of the real estate tax base assessment method is exhibited in Figure 3.

In Figure 3, case assessment has its own shortcomings, while batch assessment occupies a momentous position in the real estate market. The market method, income method, and cost method are the main tax base batch assessment methods in China. Both case assessment and batch assessment have their own advantages and disadvantages. For a wide range of real estate tax base assessment, batch assessment has certain advantages.

### 2.4. The Theory of Real Estate Tax Base Batch Assessment

Batch assessment means the process of evaluating a series of properties at a given time using standardized methods, common data, and statistical testing techniques. The application conditions of batch assessment technology cover three aspects [20]. First, it must be based on a sound and mature real estate market system. The techniques and theories of batch assessment methods, including the scope of application, are based on a well-established, mature, and regulated real estate market. Second, there must be a real estate information system, and the system has matured. The third is to have advanced scientific computer information technology support [21]. The more mature the theoretical knowledge and the more advanced the corresponding computing technology, the more mature the batch assessment technology will be, and the higher the credibility of the assessment results will be.

**Figure 4:**
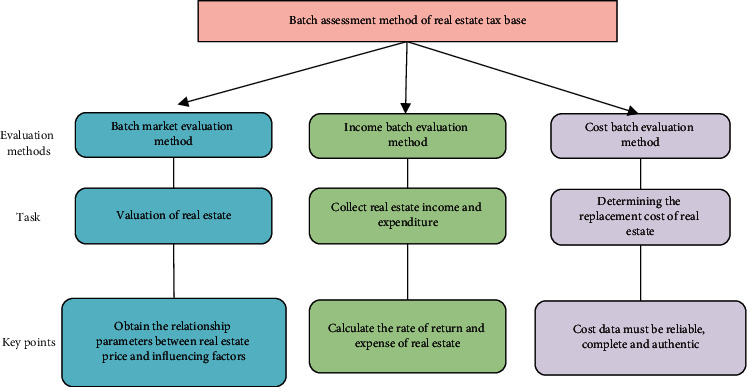
Differences of different batch assessment methods.

The traditional batch assessment method consists of the market method, the income method, and the cost method. The real estate tax base assessment method is derived from the traditional method, so the study of the traditional method plays a vital role in the real estate tax base assessment. Different batch assessment methods are distinguished, as demonstrated in Figure 4.

In Figure 4, the market method indicates the evaluation method that determines the value of the appraisal object in line with the market price of the reference object by comparing the appraisal object with the reference object. It is suitable for real estate appraisals where there are sufficient transaction cases in the real estate group. The income method denotes the conversion of the monetary value amount at a certain time point to the value amount at another time point according to the theory of the time value of money. The cost method refers to the general term for the evaluation method that determines the value of the evaluation object by taking the reconstruction or replacement cost as the basis for determining the value of the evaluation object and deducting the relevant depreciation according to the corresponding ideas. This method is mainly applicable to industrial real estate for production purposes, real estate with restricted rights or defects.

**Figure 5:**
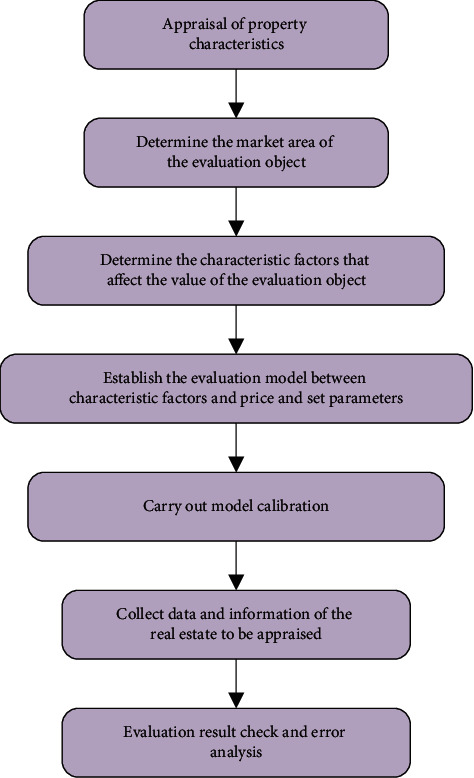
Implementation procedure of batch assessment.

Due to the different evaluation models, there are certain differences in the implementation procedure of different batch assessments. However, according to the assessment criteria, although there are differences, most of the processes are basically similar. The implementation procedure of batch assessment is mainly divided into 7 steps [22], as displayed in Figure 5.

### 2.5. DLNN

**Figure 6:**
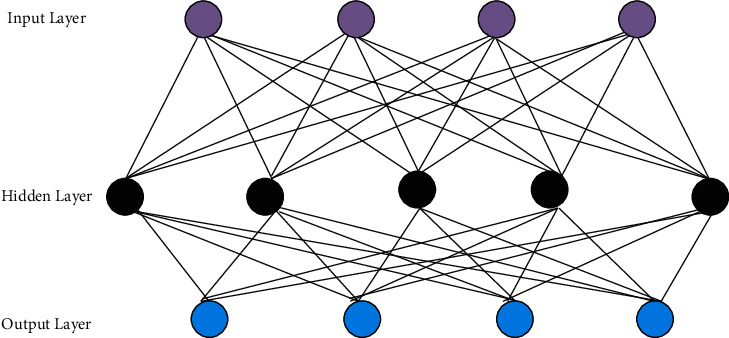
The basic model of the shallow neural network.

DL is to learn the inherent laws and representation levels of sample data, which can automatically learn data features and complete tasks such as classification and regression [23]. Its ultimate goal is to enable machines to have the same analytical capabilities and autonomous learning capabilities as humans, and to recognize data such as text and images [24]. The basic model of the shallow neural network is expressed in Figure 6.

In Figure 6, purple circles represent input layer neurons, black circles mean hidden layer neurons, and blue circles indicate output layer neurons. From top to bottom are the input layer, the hidden layer, and the output layer. There is only one hidden layer. Therefore, the initial NN model belongs to shallow learning and cannot reach the level of DL. This process can also be called the process of feature learning. The parameters of each layer obtained in the first step are further transferred to learn the parameters of the entire multi-layer model, which is similar to the random initialization of an NN. The biggest difference between DL and NNs is the first step, which is obtained by learning the structure of the input data. This value is closer to the global optimization, and the training effect is better.

**Figure 7:**
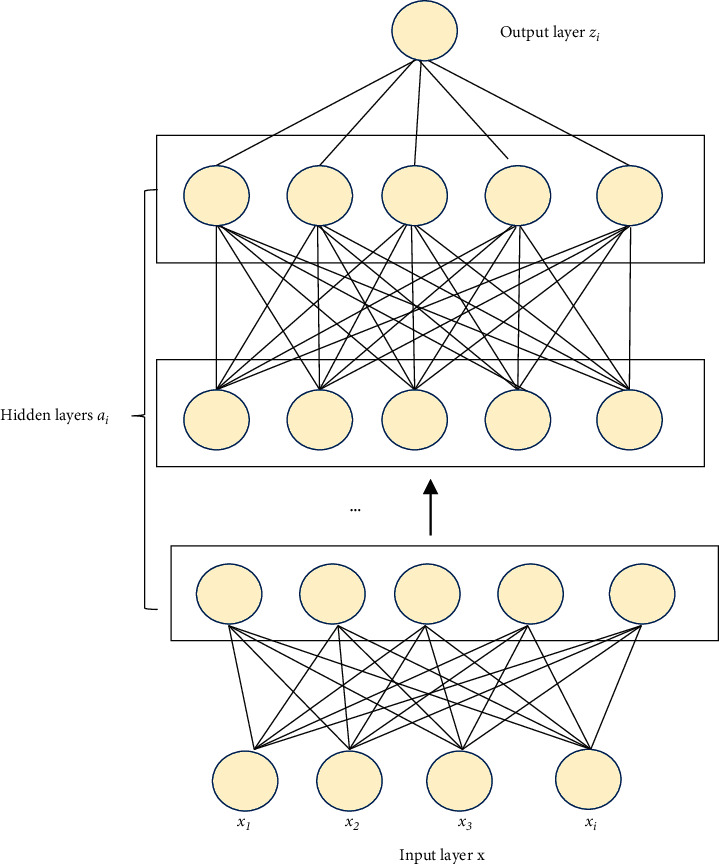
The DLNN model.

DL extracts features layer by layer by mining the feature distribution at the bottom of the data and then goes through multiple layers of processing. With multiple hidden layers, there are similarities and differences compared with the single-layer shallow model. The similarities are that the input and output layers are connected by hidden layers and the input layer is not directly connected to the output layer [25]. The structure of the DLNN model is shown in Figure 7.

In Figure 7, the unsupervised learning from the input layer to the output layer is used, that is, starting from the input layer, training layer by layer to the top layer, and training the parameters of each layer without calibration data layer by layer. This training method can be seen as an unsupervised training process.

The input layer *x*_1_ and *x*_2_ represent the sample features, the input is a real estate internal and external features, and the output is the evaluation value of the house price. *x* can be represented as

(1) $$\begin{array}{c} x={\left(x_{1},\;x_{2},\;\ldots ,x_{i}\right)}^{T}. \end{array}$$

The preactivation output is *z*_*i*_^[*l*]^, which can be expressed as

(2) $$\begin{array}{c} z_{i}^{\left[l\right]}=w_{i}^{{\left[l\right]}^{T}}+b_{i}^{\left[l\right]}. \end{array}$$

The hidden layer is *a*_*i*_^[*l*]^, and the activation output is

(3) $$\begin{array}{c} a_{i}^{\left[l\right]}=g^{\left[l\right]}\left(z_{i}^{\left[l\right]}\right). \end{array}$$

The superscript “[**l**]” means the number of layers in the NN, and the subscript signifies the number of neuron nodes [26].

Through training on large-scale data, much representative feature information is obtained. In this way, the purpose of classifying and predicting sample data is achieved, and the accuracy of classification and prediction is also improved [27].

### 2.6. DLNN-Based Real Estate Tax Base Assessment Model

In the DLNN model, the characteristic sample data *X*, parameters *w*^[1]^, *b*^[1]^ that affect the real estate value are input to the first hidden layer, and *z*^[1]^ is calculated. The specific calculation is indicated in (1):

(4) $$\begin{array}{c} z^{\left[1\right]}=w^{\left[1\right]T}X+b^{\left[1\right]}. \end{array}$$

Then *a*^[*l*]^ is calculated, the parameters *w*^[2]^, *b*^[2]^, and *a*^[*l*]^ are input to the second hidden layer together, to find *z*^[2]^ continue to find *a*^[2]^. Continuing to propagate according to such rules is called forward propagation. The calculation process is written in the following equations:

(5) $$\begin{array}{c} a^{\left[l\right]}=g\left(z^{\left[l\right]}\right),\\ z^{\left[2\right]}=w^{\left[2\right]T}a^{\left[l\right]}+b^{\left[2\right]},\\ a^{\left[2\right]}=g\left(z^{\left[2\right]}\right). \end{array}$$

By analogy, *z*^[*L*]^, *a*^[*L*]^, and $\hat{y}$ are obtained, and the computation are as follows:

(6) $$\begin{array}{c} z^{\left[L\right]}=w^{\left[L\right]T}a^{\left[L-l\right]}+b^{\left[L\right]},\\ a^{\left[L\right]}=g\left(z^{\left[L\right]}\right),\\ \hat{y}=a^{\left[L\right]}. \end{array}$$

To make the output predicted real estate value $\hat{y}$ gradually approach the real value **y**, an error loss function is defined as a measure. Its expression is written in the following equation:

(7) $$\begin{array}{c} ℒ\left(\hat{y},y\right)=\frac{1}{2}{\left(\hat{y}-y\right)}^{2}. \end{array}$$

For the NN model, it is hoped that the probability of satisfying certain conditions is expressed as follows:

(8) $$\begin{array}{c} p\left(y|x\right)=\left\{\begin{array}{cc} \hat{y}, & \left(y=1\right),\\ 1-\hat{y}, & \left(y=0\right). \end{array}\right) \end{array}$$

The above equation continues to be rewritten and can be transformed into

(9) $$\begin{array}{c} p\left(y|x\right)={\hat{y}}^{y}{\left(1-\hat{y}\right)}^{1-y}. \end{array}$$

The above results are taken logarithm on both sides, and simplified as the following equation:

(10) $$\begin{array}{c} \mathrm{Blogp}\left(y|x\right)=\mathrm{ylog}\hat{y}+\left(1-y\right)\log\left(1-\hat{y}\right). \end{array}$$

When the value of *p*(*|yx*) is larger, the loss is smaller. The cross-entropy loss function is obtained, and the expression is as follows:

(11) $$\begin{array}{c} ℒ\left(\hat{y},y\right)=-\left[\mathrm{ylog}\hat{y}+\left(1-y\right)\log\left(1-\hat{y}\right)\right]. \end{array}$$

For the overall cost function of *m* training samples, the maximum likelihood estimation (MLE) is used to obtain the likelihood function

(12) $$\begin{array}{c} P\left(x\right)=\prod_{i=1}^{m}p\left(y^{\left(i\right)}|x^{\left(i\right)}\right). \end{array}$$

The above result is taken logarithm on both sides and simplified to

(13) $$\begin{array}{c} \mathrm{logP}\left(x\right)=\sum_{i=1}^{m}\mathrm{logp}\left(y^{\left(i\right)}|x^{\left(i\right)}\right)=-\sum_{i=1}^{m}ℒ\left({\hat{y}}^{\left(i\right)},y^{\left(i\right)}\right). \end{array}$$

The goal is to minimize the cost function, and the above equation is multiplied by 1/*m* at the same time, and the cost function can be obtained [28] as illustrated in the following equation:

(14) $$\begin{array}{c} J\left(w.b\right)=\frac{1}{m}\sum_{i=1}^{m}ℒ\left({\hat{y}}^{\left(i\right)},y^{\left(i\right)}\right),=-\frac{1}{m}\sum_{i=1}^{m}\left[\mathrm{ylog}\hat{y}+\left(1-y\right)\log\left(1-\hat{y}\right)\right]. \end{array}$$

### 2.7. Experimental Testing and Data Processing

China's residential real estate accounts for the largest proportion of all real estate, with relatively similar structural characteristics and high homogeneity. The data comes from the Internet sales platform, that is, a third-party website. The name, building age, floor, number of rooms, orientation, building area, decoration status, property status, and other characteristics of each community are obtained from the second-hand housing transaction website. To meet the training needs of deep neural network (DNN), the second-hand housing in Hefei is selected as the research object, and based on the price index of real estate tax base assessment, 10 second-hand housing transaction data are selected as the sample data. According to the evaluation index system, the quantified values of 7 indicators such as the number of rooms and area involved are input from the input layer to the neural network model, and the output layer outputs the predicted value of the real estate transaction price (unit price). The basic situation of the sample is exhibited in Table 1.

The data in Table 1 represent the source code of the program. The original data is collected through the network, and there may be irregularities. Some data are missing, and the mean value of similar data is used to make up. Due to the difference in scale, a large range of data will cause the problem that the model training is not easy to converge, affecting the entire learning effect. Therefore, the data set needs to be normalized to facilitate the speed of training and optimization of the network. The average value of the sample is calculated first, the average value is subtracted from the sample, and the data is moved to the center of the coordinate axis. Then the variance of the sample is calculated to standardize the variance.

## 3. Model Validation and Result Discussion

### 3.1. Reliability Test of Models

**Figure 8:**
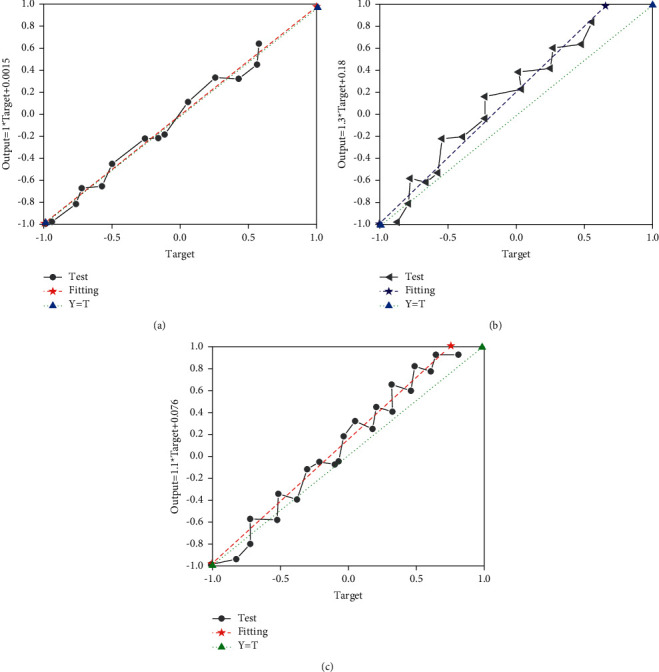
Regression results of DLNN. (a) Training *R*=0.99996; (b) validation *R*=0.96684; (c) all *R*=0.95708.

The error is used to test whether the model is reliable.  Figure 8 denotes the error test of DLNN regression results.

In Figure 8, the regression effect of the output value under different coefficients of determination *R* is relatively good. The test value is close to the fitted value, which can be well distributed around the fitting curve. The coefficient of determination *R* is close to 1, showing that the fitting effect is better. All discrete points can be distributed around the fitted line, with small errors and fast convergence. It means that the DLNN model has a good degree of fit and high reliability.

### 3.2. Comparison with Results from Other Machine Learning (ML) Algorithms

**Figure 9:**
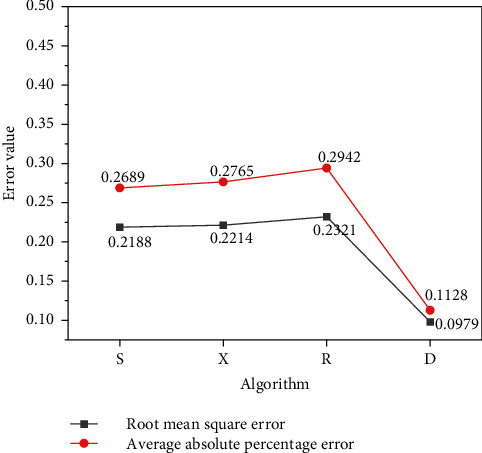
Comparison of prediction errors of different algorithm models.

To study the advantages and disadvantages of DLNN prediction compared with other algorithms, for the same data, three algorithms of Stacking integrated model, Extreme Gradient Boosting (XGboost) forecast, and ridge regression prediction are used to make predictions and compared with DLNN. According to the same steps, sample 1 data is selected for training, and the comparison results of the root mean square error (RMSE) and the mean absolute percentage error (MAPE) predicted by different algorithm models are exhibited in Figure 9.

In Figure 9, the error results obtained by different learning models are different and the RMSE is compared. *S* stands for the Stacking ensemble model, *X* refers to XGboost prediction, *R* means ridge regression prediction, and *D* denotes the DL model. The error values of the other three algorithms are all above 0.2, while the RMSE value of the DL model algorithm is at least 0.0979. The value of RMSE is significantly lower than other algorithms. Using different algorithms to calculate MAPE, it can also be found that the MAPE of DL is 0.1128. The error of the other three algorithms is above 0.25, and the error is relatively large. The error value of the DL model algorithm is relatively small, and the error value of the test result is the smallest. It manifests that the DL model can more accurately approach the real value, and the DLNN model has more advantages than other algorithms.

### 3.3. Accuracy Analysis of Models

**Figure 10:**
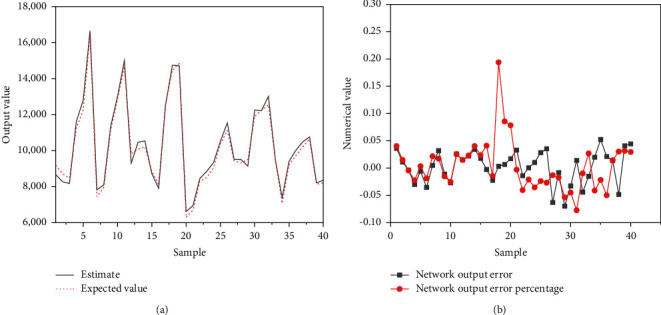
Validation results of the prediction accuracy of the model. (a) Comparison of predicted output and actual output; (b) forecast error and error percentage.

The accuracy of the model is verified, and after 40 iterations, the analysis results can reach the predetermined target. The validation results of the prediction accuracy of the model are displayed in Figure 10.

In Figure 10, the predicted output value of the model is close to the actual value, and the accuracy is high. After iterative training of samples, DLNN can reach the predetermined error target. After the model undergoes continuous training iterations, the predicted results of the samples can be fitted with the actual results. In Figure 10(b), the prediction error is relatively small and the error value remains within 0.05. Except for individual values, the prediction error percentages are kept within a controllable range. The above can prove that the model is successfully trained, the training results have small errors, and the convergence is fast. The designed model has high accuracy.

### 3.4. Analysis of Test Training of Samples

**Figure 11:**
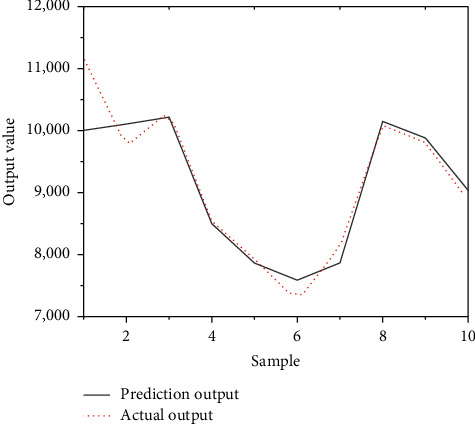
Comparison of predicted and actual values for the test sample.

10 samples were selected for the experiment, the quantitative results of the index system of the test samples were input into the model, the predicted and actual values of the experimental samples were obtained, and the predicted and actual values of the samples were analyzed.  Figure 11 demonstrates the comparison result between the predicted value of the test sample and the actual value.

In Figure 11, when testing the samples, except that the predicted values of samples 2 and 6 are higher than the actual values, and there is a large error between the two, the errors between the predicted values and the actual values of other samples are small, and the two values are relatively close. The predicted values of the 8 samples can be well fitted with the actual values, the predicted prices are close to the actual prices, and the model's price estimates are accurate. Overall, the evaluation effect of the real estate tax base is better.

### 3.5. Analysis of Test Error Values for Samples

**Figure 12:**
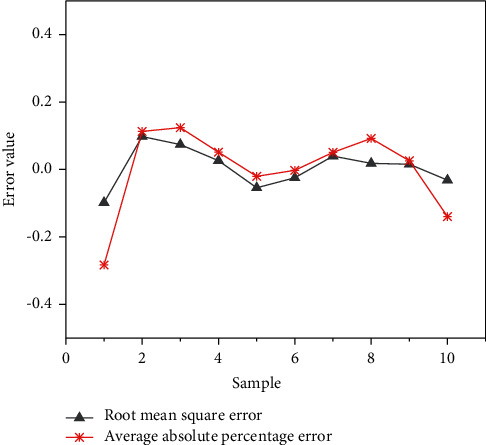
Error comparison between test value and actual value.

The test value and actual value of the obtained sample are subjected to error analysis.  Figure 12 indicates the results calculated with RMSE and MAPE, respectively.

In Figure 12, although there is an error in the test, the error can be controlled within the range of 0.4. After continuous iteration of DNN, the error between the predicted price and the actual transaction price is very small, illustrating that the DLNN model has good performance in function approximation and better accuracy. After many iterations of training, a more accurate estimate can be obtained when the test value of samples is finally performed.

### 3.6. Result Analysis of Normalized Processing of Test Data

**Figure 13:**
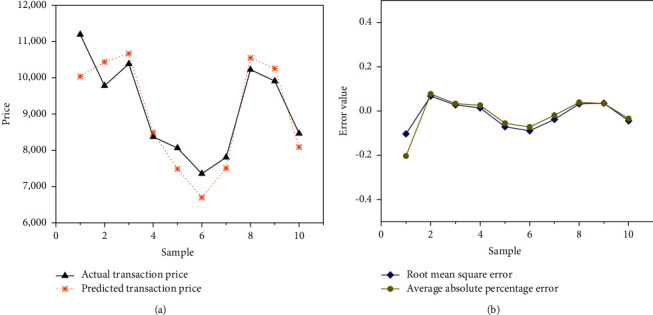
Comparison of error between test value and actual value of transaction price. (a) The comparison between the actual transaction price and the network predicted price; (b) the comparison between the RMSE and the MAPE of the transaction price.

After the test results are normalized, the error analysis is carried out with the actual transaction value. The comparison result between the test value and the actual value of the transaction price is shown in Figure 13.

In Figure 13, the trained model can well simulate the relationship between the factors affecting real estate prices, and the predicted price is relatively close to the actual price. This testifies that as long as batch samples are used to train the model in advance and accurate training functions and parameters are selected, the model can output large batches of relatively accurate tax base assessment prices. Therefore, through experimental training, it can be proved that the model has excellent performance in tax base assessment and can meet the requirements of efficient batch assessment. Completing a huge amount of work in a limited time not only reduces costs but also improves work efficiency. The training results have small errors and fast convergence.

Compared with other machine learning algorithms, the proposed DLNN has a relatively small RMSE and MAPE value in the prediction error analysis of training samples. The predicted value of the DL model is closer to the real value in terms of prediction and has advantages over other algorithms. Through sample test training, the predicted value and the actual value can be well fitted, the predicted price is close to the actual price, and the model has a better effect on the evaluation of the real estate tax base. Through the continuous iteration of the DLNN, the error between the predicted price and the actual transaction price is small, and the DLNN model has good performance in function approximation [29].

## 4. Conclusion

First, the concept of real estate tax base assessment is explained, and the similarities between real estate tax base assessment and real estate appraisal are compared. The index factors of real estate tax base assessment are analyzed, focusing on the factors that affect real estate appraisal. Then, the general methods of real estate tax base assessment are compared and analyzed, and the batch assessment method is proposed to have certain advantages. Next, the theory of real estate tax-based batch assessment is further studied, and the implementation procedures of batch assessment are described in detail by comparing different batch assessment methods. The DLNN model is applied to the real estate tax base assessment, and a real estate tax base assessment model in accordance with DLNN is constructed, and the reliability and accuracy of the model are analyzed. At last, compared with other algorithms, the advantages of DLNN's real estate tax base assessment model are highlighted, and the model is used to test samples and analyze samples and errors. The results manifest that (1) by testing the reliability of the model, it is found that the DLNN model has a good degree of fit. The test value is close to the fitted value, and it can be well distributed around the fitting curve. It can be seen that the DLNN model has a good degree of fit and high reliability; (2) compared with the other three learning algorithms, the RMSE of the DL model algorithm is the smallest, which is significantly lower than other algorithms, and the MAPE is also the smallest. It illustrates that the DL model algorithm has the smallest prediction error, and the evaluation prediction results are closer to the true value; (3) after verifying the accuracy of the model, it denotes that after continuous training iterations, the sample prediction results can be fitted with the actual results. The error of training results is small and the convergence speed is fast. The designed model has high accuracy; (4) the DLNN model has good accuracy in function approximation, and the error is controllable and small. To sum up, the real estate tax base assessment model based on DLNN has higher accuracy than other algorithms, and its prediction error is the smallest, which can meet the requirements of efficient batch assessment of the real estate tax base. It is expected to complete a huge amount of work in a limited time, which can effectively improve work efficiency.

## Figures and Tables

**Table 1:** The basic conditions of the test samples.

Sample	Number of rooms	Floor	Building age	Orientation	Housing area	Environment	Property	Property price
1	3	5	5	5	2	4	4	11195
2	4	3	5	5	4	3	3	9782
3	5	5	5	4	5	4	4	10390
4	5	3	3	5	3	4	4	8375
5	4	5	5	5	2	3	3	8065
6	5	3	5	5	5	4	4	7355
7	5	5	5	5	4	4	4	7805
8	6	3	3	5	4	5	3	10226
9	6	3	5	5	5	4	4	9922
10	4	3	5	5	3	4	3	8466

## Data Availability

The data used to support the findings of this study are included within the article.

## References

[1] Parrillo N. R. (2020). A Critical Assessment of the Originalist Case Against Administrative Regulatory Power: New Evidence from the Federal Tax on Private Real Estate in the 1790s. *Yale LJ*.

[2] Pavlov K., Pavlova O., Pavlova V. (2019). Integral indicators based on competitiveness capacity characteristics of regional real estate markets of Ukraine. *Journal of Competitiveness*.

[3] Ghosh C., Liang M., Petrova M. T. (2020). The effect of fair value method adoption: evidence from real estate firms in the EU. *The Journal of Real Estate Finance and Economics*.

[4] Wei C., Fu M., Wang L., Yang H., Tang F., Xiong Y. (2022). The research development of hedonic price model-based real estate appraisal in the era of big data. *Land*.

[5] Bin J., Gardiner B., Liu Z., Li E. (2019). Attention-based multi-modal fusion for improved real estate appraisal: a case study in Los Angeles. *Multimedia Tools and Applications*.

[6] Guo Y., Lin S., Ma X., Bal J., Li C. T. (2019). Homogeneous Feature Transfer and Heterogeneous Location Fine-Tuning for Cross-City Property Appraisal Framework. *Data Mining. AusDM 2018. Communications in Computer and Information Science*.

[7] Acci L. (2019). Quality of urban area, distance from city centre, and housing value. *Case study on real estate values in Turin. Cities*.

[8] Wang D., Li V. J. (2019). Mass appraisal models of real estate in the 21st century: a systematic literature review. *Sustainability*.

[9] Chen Y., Hu S., Mao H., Deng W., Gao X. (2020). Application of the best evacuation model of deep learning in the design of public structures. *Image and Vision Computing*.

[10] Li Y., Zhao J., Lv Z., Li J. (2021). Medical image fusion method by deep learning. *International Journal of Cognitive Computing in Engineering*.

[11] Chen D., Wawrzynski P., Lv Z. (2021). Cyber security in smart cities: a review of deep learning-based applications and case studies. *Sustainable Cities and Society*.

[12] Lv Z., Qiao L. (2020). Deep belief network and linear perceptron based cognitive computing for collaborative robots. *Applied Soft Computing*.

[13] Zhang H., Li Y., Lv Z., Sangaiah A. K., Huang T. (2020). A real-time and ubiquitous network attack detection based on deep belief network and support vector machine. *IEEE/CAA Journal of Automatica Sinica*.

[14] Zhang P. (2021). Research on the reform of real estate taxation in the personal housing ownership link. *Proceedings of Business and Economic Studies*.

[15] Kalkuhl M., Fernandez Milan B., Schwerhoff G., Jakob M., Hahnen M., Creutzig F. (2018). Can land taxes foster sustainable development? An assessment of fiscal, distributional and implementation issues. *Land Use Policy*.

[16] Renigier-Biłozor M., Janowski A., d’Amato M. (2019). Automated valuation model based on fuzzy and rough set theory for real estate market with insufficient source data. *Land Use Policy*.

[17] Battisti F., Campo O. (2019). A methodology for determining the profitability index of real estate initiatives involving Public–Private partnerships. A case study: the integrated intervention programs in rome. *Sustainability*.

[18] Baldauf M., Garlappi L., Yannelis C. (2020). Does climate change affect real estate prices? Only if you believe in it. *Review of Financial Studies*.

[19] Poursaeed O., Matera T., Belongie S. (2018). Vision-based real estate price estimation. *Machine Vision and Applications*.

[20] Qiang Q. (2019). Analysis of debt-paying ability of real estate enterprises based on fuzzy mathematics and K-means algorithm. *Journal of Intelligent and Fuzzy Systems*.

[21] Moutinho L. F., Moura F. R., Silvestre R. C., Romao Dumaresq A. S. (2021). Microbial biosurfactants: a broad analysis of properties, applications, biosynthesis, and techno economical assessment of rhamnolipid production. *Biotechnology Progress*.

[22] Shi D., Guan J., Zurada J., Levitan A. S. (2022). Predicting home sale prices: a review of existing methods and illustration of data stream methods for improved performance. *WIREs Data Mining and Knowledge Discovery*.

[23] Moen E., Bannon D., Kudo T., Graf W., Covert M., Van Valen D. (2019). Deep learning for cellular image analysis. *Nature Methods*.

[24] Wang S., Yang D. M., Rong R., Zhan X., Xiao G. (2019). Pathology image analysis using segmentation deep learning algorithms. *American Journal Of Pathology*.

[25] Li C. H., Fink C., Lippold J. C., Jinschek J. R. (2020). Identification of interdendritic phases in Ni30Cr weld metal with additions of tantalum and molybdenum using electron diffraction pattern and high-resolution scanning transmission electron microscopy image analysis. *Materials Characterization*.

[26] Zappone A., Di Renzo M., Debbah M. (2019). Wireless networks design in the era of deep learning: model-based, AI-based, or both. *IEEE Transactions on Communications*.

[27] Ozbayoglu A. M., Gudelek M. U., Sezer O. B. (2020). Deep learning for financial applications: a survey. *Applied Soft Computing*.

[28] Schmidt J., Marques M. R. G., Botti S., Marques M. A. L. (2019). Recent advances and applications of machine learning in solid-state materials science. *Npj Computational Materials*.

[29] Beimer J., Francke M. (2019). Out-of-sample house price prediction by hedonic price models and machine learning algorithms. *Real Estate Research Quarterly*.

